# Feeling the heat: developmental and molecular responses of wheat and barley to high ambient temperatures

**DOI:** 10.1093/jxb/eraa326

**Published:** 2020-07-16

**Authors:** Catherine N Jacott, Scott A Boden

**Affiliations:** 1 Department of Crop Genetics, John Innes Centre, Colney Lane, Norwich, UK; 2 School of Agriculture, Food and Wine, Waite Research Institute, Waite Research Precinct, University of Adelaide, Glen Osmond, SA, Australia; 3 University College Dublin, Ireland

**Keywords:** Barley, cereals, high temperature, reproductive development, thermal resilience, wheat

## Abstract

The increasing demand for global food security in the face of a warming climate is leading researchers to investigate the physiological and molecular responses of cereals to rising ambient temperatures. Wheat and barley are temperate cereals whose yields are adversely affected by high ambient temperatures, with each 1 °C increase above optimum temperatures reducing productivity by 5–6%. Reproductive development is vulnerable to high-temperature stress, which reduces yields by decreasing grain number and/or size and weight. In recent years, analysis of early inflorescence development and genetic pathways that control the vegetative to floral transition have elucidated molecular processes that respond to rising temperatures, including those involved in the vernalization- and photoperiod-dependent control of flowering. In comparison, our understanding of genes that underpin thermal responses during later developmental stages remains poor, thus highlighting a key area for future research. This review outlines the responses of developmental genes to warmer conditions and summarizes our knowledge of the reproductive traits of wheat and barley influenced by high temperatures. We explore ways in which recent advances in wheat and barley research capabilities could help identify genes that underpin responses to rising temperatures, and how improved knowledge of the genetic regulation of reproduction and plant architecture could be used to develop thermally resilient cultivars.

## Teaser

We outline recent advancements in our understanding of the reproductive developmental traits and genetic pathways underpinning responses of wheat and barley to high ambient temperatures, and propose new strategies for developing thermotolerant cultivars.

## Introduction

Global agriculture is challenged by increasing populations and climate change. In particular, cereals including bread wheat (*Triticum aestivum*), durum wheat (*Triticum turgidum*), and barley (*Hordeum vulgare*) are major sources of human nutrition for which grain yield and quality are significantly affected by high temperature (Wardlaw *et al.*, 1989; Wallwork *et al.*, 1998; Battisti and Naylor, 2009; Lobell *et al.*, 2011). For example, a temperature increase of 2 °C reduces global wheat yields by 11%; based on 2017 production, this equates to a yield loss of 84.8 Mt, which is higher than the total amount of wheat produced annually in Northern Europe (Zaveri and Lobell, 2019). Climate models predict that cereals will be exposed to higher average temperatures and more frequent extreme heat stress in the future (Lobell *et al.*, 2011). Coupled with the requirements to increase yields by 60–70% over the next 50 years in order to maintain global food security, the effects of high temperature on development and the molecular mechanisms that underpin downstream responses should be investigated further to help generate thermotolerant germplasm (Jaggard *et al.*, 2010).

To understand the effects of a warming climate, it is important to consider stages of development that are critically influenced by higher temperatures. The life cycle of cereals progresses through distinct growth phases: vegetative, reproductive (inflorescence/spike development), anthesis (flowering), grain set, and senescence. The transition between these growth phases is dependent on developmental programmes that are activated and modulated by environmental and endogenous stimuli (Huijser and Schmid, 2011). Temperature and photoperiod (the duration of light during the day) are critical environmental signals used as seasonal cues to coordinate developmental transitions (Fjellheim *et al.*, 2014). During crop domestication, allelic variation for genes that regulate photoperiod and thermal-dependent initiation of flowering has broadened cultivation ranges, such that flowering and grain development occur under optimal conditions (Worland, 1996; Worland *et al.*, 1998; Jung and Müller, 2009). Although cereals are widely adapted to grow in many areas of the world (Cockram *et al.*, 2007), the late reproductive phases of development are particularly susceptible to temperature stress (Porter and Gawith, 1999; Wollenweber *et al.*, 2003). The physiological response of cereals to high temperatures during late reproductive stages has been well studied, and our understanding of the molecular processes that underpin responses to warming temperatures is improving as we learn more about the genes that coordinate reproductive development. Further advances in our understanding of these genes will benefit from improved genetic knowledge and technical capabilities in wheat and barley research (Krasileva *et al.*, 2017; Mascher *et al.*, 2017; IWGSC, 2018; Ramírez-González *et al.*, 2018; Watson *et al.*, 2018).

Here, we discuss the effects of high temperature on the reproductive development of wheat and barley, including traits such as flowering time, inflorescence development, and grain filling. We consider exposure to high temperatures—average, acute, and night-time—as well as the response of other cereal crops to warmer conditions. We link historical phenology-based studies with more recent work investigating the genetic regulation of high-temperature responses, including the potential to learn from outcomes of research using model plants. In doing so, we set the stage for future research that aims to improve the thermal resilience of wheat and barley.

## Temperature effects on reproductive development

Wheat and barley are temperate, facultative long-day plants that flower more rapidly under long-day photoperiods and are characterized by two growth types: winter and spring. Winter types show accelerated flowering after prolonged exposure to the cold temperatures of winter—a process known as vernalization—which coordinates floral development and delays the cold-sensitive reproductive growth phases until spring (Purvis, 1934; McKinney, 1940; Trevaskis, 2010). In contrast, spring types do not require a period of cold to flower (Trevaskis, 2010; Gol *et al.*, 2017). Responses to these seasonal cues align flowering with the favourable conditions of spring, enabling the completion of fertilization and grain production before the onset of hot and dry conditions of summer (Fjellheim *et al.*, 2014). The growth habit of the plant also influences its response to high temperatures (Craufurd and Wheeler, 2009; Dixon *et al.* 2019). For example, in field experiments using supplementary heating, spring wheat plants exposed to temperatures between 0 °C and 40 °C flowered significantly earlier with increasing temperature (White *et al.*, 2011). In winter wheat, however, high-temperature treatments during or post-vernalization delay early and late stages of reproductive development, with the flowering of some cultivars occurring later at 25 °C than at 11 °C or 18 °C (Dixon *et al.*, 2019).

**Fig. 1.**
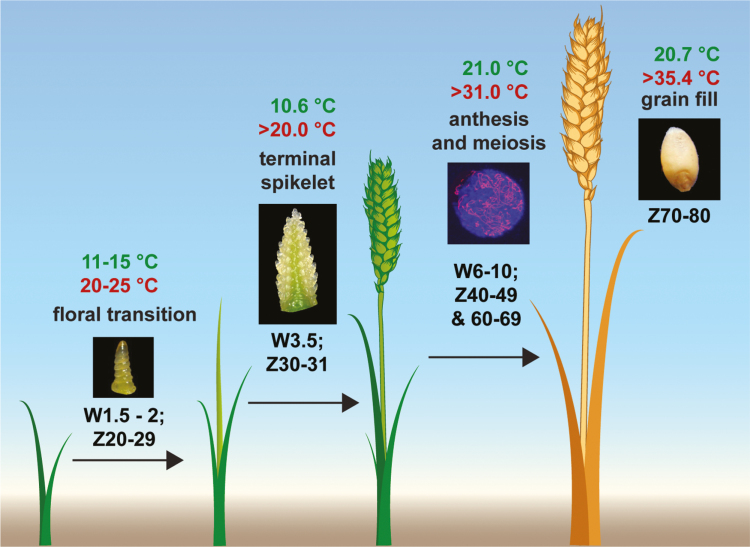
Optimum and maximum temperatures for the reproductive growth stages in wheat. Optimum temperatures are in green, and maximum temperatures (temperatures higher than which are damaging) are in red for key developmental stages of wheat and barley that are vulnerable to high temperatures. Insert images show a double ridge apex, terminal spikelet apex, prophase I meiotic cell with DAP1 (blue) and *Ta*ASY1 (red), and grain at maturity (from left to right). The respective Zadoks (Z; Zadoks *et al.*, 1974) and Waddington (W; Waddington *et al.*, 1983) scales for each growth stage are indicated.

As expected for plants that have adapted to the progression of seasons from winter into spring, the ideal growth temperatures for early developmental stages are cooler than those of later stages. For example, the optimum temperatures for wheat at the terminal spikelet stage, anthesis, and grain filling, as determined from 65 published studies, are 10.6, 21.0, and 20.7 °C, respectively (Porter and Gawith, 1999; Fig. 1). As a consequence, the temperature at which thermal stress occurs is dependent on the developmental stage (Slafer and Rawson, 1995). During early wheat inflorescence development, when the number of spikelets is being determined (the terminal spikelet stage), an increase in temperature from 10 °C to 19 °C accelerates reproductive development, whereas temperatures >20 °C delay terminal spikelet initiation and reduce the number of spikelet primordia (Slafer and Rawson, 1995; Porter and Gawith, 1999). Similar results have been reported in spring and winter barley, where an increase in ambient temperature from 20 °C/16 °C (day/night) to 28 °C/24 °C and from 15 °C to 25 °C, respectively, delayed inflorescence development, and reduced floret number and grain per spike (florets are equivalent to spikelets in these cases, as the research used two-rowed cultivars) (Hemming *et al.*, 2012; Ejaz and von Korff, 2017). The decrease in spikelet and floret number that accompanies delayed inflorescence development under high temperatures somewhat contradicts expectations, as slower development of the inflorescence is normally associated with the production of more spikelets (e.g. under short-day photoperiods). These results suggest that high temperatures inhibit both inflorescence and spikelet development during early reproductive stages of barley and wheat.

The average optimum temperature for anthesis in wheat is 21.0 °C, and temperatures higher than 31 °C limit the success of pollination (Porter and Gawith, 1999; Fig. 1). Exposure of wheat and barley to high temperatures around anthesis results in a non-recoverable reduction of yield associated with floret infertility, due to adverse effects on ovary development and pollen viability, and a decreased rate of grain fill (Saini *et al.*, 1983; Stone and Nicolas, 1996; Ferris *et al.*, 1998; Wallwork *et al.*, 1998; Sakata *et al.*, 2000). For example, field-based analyses showed that short periods of high temperature (31.8 °C) immediately before anthesis significantly decreased grain mass, and Australian spring wheat varieties were sensitive to high temperatures (24 °C) within 3 d following anthesis, resulting in smaller grains at maturity (Chowdhury and Wardlaw, 1978; Stone and Nicolas, 1995*c*; Savin *et al.*, 1999). However, at 8 d post-anthesis, high temperatures had little effect on grain mass or the number of deformed grains (Stone and Nicolas, 1995*a*, *b*). Short periods of moderately high temperatures are also detrimental to meiosis, with a treatment of 30 °C for 20–24 h preventing the progression of pollen mother cells through early meiotic stages, reducing pollen viability and grain number (Draeger and Moore, 2017). These outcomes highlight the importance of ongoing research investigating the effect of high temperatures on gamete development and fertilization, particularly because plants are more likely to encounter heat stress during late reproductive stages.

Grain development is another stage of the life cycle that is likely to experience thermal stress (Fig. 1). Exposure to high ambient temperatures during grain filling has an impact on yield by reducing grain number, weight, and quality (Stone and Nicolas, 1994; Wardlaw and Moncur, 1995; Gibson and Paulsen, 1999; Pradhan *et al.*, 2012). The average optimum temperature for grain fill in wheat is 20.7 °C, and temperatures higher than 35.4 °C are damaging (Porter and Gawith, 1999; Shirdelmoghanloo *et al.*, 2016). For example, plants grown in glasshouses and treated to temperatures of 40 °C during grain fill significantly reduced grain quality by decreasing protein accumulation (Stone and Nicolas, 1996). Regarding grain filling, high temperatures can increase assimilate supply; however, the duration of grain filling is reduced without a compensatory increase in the rate of grain fill, resulting in significantly lower yield (Tashiro and Wardlaw, 1989; Wardlaw and Moncur, 1995; Wardlaw *et al.*, 1995; Lobell *et al.*, 2012; Pradhan *et al.*, 2012). The accelerated development of grains at high temperatures is consistent with transcriptome analyses, where treatments at 28, 37, and 42 °C advanced and compressed the expression of genes that regulate metabolic processes of grain development in wheat and barley, relative to control conditions (Altenbach and Kothari, 2004; Wan *et al.*, 2008; Mangelsen *et al.*, 2011).

Taken together, these analyses highlight the importance of investigating a broad range of traits and temperatures when considering the impact of a warming climate on wheat and barley reproduction. These detailed analyses of late reproductive development have set the stage for molecular experiments to now be performed, which aim to understand the genes that coordinate responses to warmer growth conditions.

### Interactions between temperature and photoperiod

Photoperiod has an important role in determining the effects of high ambient temperature on reproductive development. For example, an ambient temperature of 30 °C delays spikelet initiation in barley in a photoperiod-dependent manner (Aspinall, 1969). In winter barley and fast-flowering barley lines (containing a mutation in *EARLY FLOWERING3*, *ELF3*), an increase in temperature from 15 °C to 25 °C resulted in rapid progression through reproductive development in long days but inhibited early developmental stages when plants were grown under short daylengths (Hemming *et al.*, 2012). In long-day photoperiods, the increase in temperature from 15 °C to 25 °C coincided with the production of more spikes per plant. However, plants produced fewer primordia and florets per inflorescence, resulting in fewer florets per plant at 25 °C. In short days, the increase in temperature from 15 °C to 25 °C resulted in smaller shoot apices and fewer primordia (Hemming *et al.*, 2012). In wheat, high temperatures caused a similar response under short daylengths, with early inflorescence development being delayed in isogenic lines containing photoperiod-insensitive or null alleles of *Photoperiod-1* (*Ppd-1*) and a weak vernalization response (Rawson and Richards, 1993; Hemming *et al.*, 2012). Under long days, high temperatures reduced the duration of time to double ridge and slowed the production of spikelet primordia, but plants flowered earlier at 25 °C than at 15 °C, suggesting that warmer conditions accelerate later developmental stages (Rawson and Richards, 1993). Similar results were found in a collection of spring and winter photoperiod-sensitive wheat lines containing different alleles of *Ppd-1* and *VERNALIZATION1* (*VRN1*), with warmer temperatures delaying reproductive development, particularly under short daylengths (Kiss *et al.*, 2017). Together, these observations suggest that high temperatures inhibit reproductive development in non-inductive photoperiods, especially during early developmental stages, but accelerate reproduction under inductive daylengths. The interaction between temperature and photoperiod may indicate a conserved adaptation of temperate cereals to warmer conditions, which is dependent on progression through the season. For example, the interaction may help plants recognize that unseasonably warm temperatures during winter and early spring should be ignored as cues to flower, while warmer conditions during late spring and early summer signal that more hot and dry conditions are likely, promoting fertilization and grain fill before the onset of more extreme conditions.

### Effects of chronic and acute high-temperature treatments on yield components

Climate projections suggest that extreme temperature events will become more intense and more frequent (Meehl *et al.*, 2007; IPCC, 2013). In the field, stress events include short treatments of high temperatures (acute temperature stress) as well as sustained periods of warmer than average temperatures (chronic high-temperature stress). With both rising temperatures and frequency of acute high temperatures identified as significant threats to crop production (Challinor *et al.*, 2005), it is important to consider the impact of both chronic and acute high temperatures for accurate prediction of crop performance under climate change scenarios (Barlow *et al.*, 2015).

In wheat, the effects of acute and chronic warm temperatures are particularly significant during reproductive development, with treatments during early and late stages influencing various components of yield. For example, chronic exposure to warmer temperatures (2.6–5.4 °C above ambient) during early reproductive development (double ridge and terminal spikelet stages) of field-grown winter wheat decreased yield by reducing the number of grain-producing spikelets formed per inflorescence—the effect on reduced grain numbers was partially compensated by an increase in grain weight (Johnson and Kanemasu, 1983). Exposure to short or extended periods of high temperature (30 °C) later in development—including stages up to immediately prior to anthesis—dramatically reduced grain numbers and grain weight (Saini and Aspinall, 1982; Ugarte *et al.*, 2007). Acute exposure to extremely high temperatures (40 °C) 2 weeks after anthesis and prolonged treatment of moderately high temperatures (21, 27, or 30 °C) 3 weeks after anthesis both led to a reduction in yield by decreasing grain weight (Stone *et al.*, 1995). Four day treatments at 40 °C and prolonged exposure to 27 or 30 °C significantly reduced grain weight relative to control conditions; each 1 °C rise perturbed grain weight by 2.5% (Stone *et al.*, 1995). In comparison, identical treatments during late developmental stages reduced grain weight without influencing grain number (Johnson and Kanemasu, 1983), and exposure to short periods of high temperature (30 °C for 3 d before anthesis) resulted in reduced grain set without a compensatory increase in grain weight (Saini and Aspinall, 1982). Taken together, these studies show that the effects of acute treatment of high temperatures and prolonged chronic stress under moderate heat merit equal investigation when studying the effects of warmer conditions on cereal productivity (Balla *et al.*, 2019).

Heat acclimation has been identified as a possible strategy to mitigate yield losses of wheat caused by high-temperature stress (Stone and Nicolas, 1995*b*). One study investigated whether a sudden rise in temperature (20 °C to 40 °C) resulted in a more significant reduction in individual kernel mass than a gradual rise over the same temperature range (6 °C h^–1^), using wheat cultivars differing in heat tolerance. For the heat-sensitive genotype, the reduction of individual grain mass following sudden heat stress was higher than that resulting from gradual heat stress. On the other hand, for the heat-tolerant genotype, there was no significant difference in individual kernel mass of plants treated abruptly versus those warmed more gradually (Stone and Nicolas, 1995*b*). These results support the investigation of both chronic and acute heat stress on reproductive development, especially when considering cultivar-specific responses and the different ways plants are exposed to high temperatures in the mega-environments where wheat and barley are grown (Sonder, 2016).

### High night-time temperatures

Climate models predict more substantial increases in night-time temperatures compared with daytime temperatures. In the past 100 years, global daily minimum (night-time) temperatures increased >2-fold compared with increases in daily maximum (daytime) temperatures (Easterling *et al.*, 1997). Critical developmental stages are differentially sensitive to minimum and maximum temperatures, and historical yields of wheat are more strongly correlated with daily minimum temperatures compared with maximum temperatures (Lobell and Ortiz-Monasterio, 2007). Researchers and breeders should, therefore, consider the responses of wheat and barley to both increasing daytime and night-time temperatures in the pursuit of generating thermally resilient cultivars.

In wheat grown under controlled-environment conditions, exposure to high night-time temperature (HN) of ≥20 °C and constant daytime temperature (24 °C) decreased spikelet fertility, grains per spike, and grain size, compared with the control (14 °C night-time temperature; Prasad *et al.*, 2008*b*). Furthermore, exposure to HN decreased the duration of grain filling (Prasad *et al.*, 2008*b*), and each 1 °C increase in night temperatures reduced yield of winter wheat by 6%, and 4–7% in spring wheat and barley (García *et al.*, 2015; Hein *et al.*, 2019). Similar observations were made in the field using barley and wheat grown under ambient daytime temperatures and HN; HN reduced grain yields, which was associated with accelerated reproductive development and a shorter critical period for grain filling (García *et al.*, 2015). The molecular processes that influence the response to HN are unknown; however, the effect of HN may be linked with decreases in the amount of photoassimilates available for plant growth and grain fill, caused by higher respiration at night (Garcia *et al.*, 2016; Impa *et al.*, 2019; Sadok and Jagadish, 2020). Similarly, it has been proposed that the HN treatment may depress photosynthesis-dependent processes, accelerating leaf senescence and reducing the effective period of light interception by the plant, which would reduce photoassimilate distribution to the grain (Shirdelmoghanloo *et al.*, 2016; Lesjak and Calderini, 2017; Sadok and Jagadish, 2020). Dissection of the molecular responses that occur under warm night-time temperatures will therefore benefit from a multidisciplinary approach that includes physiology, developmental biology, and modelling.

Studies of winter wheat have investigated differences between exposure to HN, high daytime (HD), and both high daytime and night-time temperatures (HDN). One study suggested that the impact of HN and HD was similar on all reproductive traits; for example, both HN and HD during anthesis caused damage of comparable magnitude. However, more substantial decreases in seed set, grain number, and grain yield per inflorescence were observed at HDN, compared with either HD or HN (Narayanan *et al.*, 2015). In contrast, a study under field conditions suggested that there was no significant difference in yield and biomass between plants exposed to HN versus HDN (Fang *et al.*, 2015). The outcomes of these studies highlight the importance of considering high minimum as well as maximum temperatures in assessing the response of wheat and barley to a warming climate, particularly during late reproductive stages.

### Responses of other cereals to high temperatures

In contrast to temperate cereals (wheat, barley, oat, and rye), tropical crops such as corn, rice, and sorghum are short-day plants that are often cultivated in warmer climates. Data describing responses of maize and rice to high temperatures, particularly during later developmental stages, may be used to improve thermal resilience of wheat and barley (Caddel and Weibel, 1972; Russell and Stuber, 1983; Izawa *et al.*, 2000; Leff *et al.*, 2004).

Studies comparing the effects of temperature on reproductive development between wheat and other cereals have shown that the optimum growth temperatures for corn, rice, and sorghum are higher than for wheat. For example, one study showed a significant difference between wheat (15/10 °C) and sorghum (27/22 °C) in optimum temperatures for kernel dry weight (Machado and Paulsen, 2001). Similar to wheat and barley, corn, rice, and sorghum are sensitive to high-temperature treatments—particularly during reproductive development, anthesis, and grain fill—resulting in decreased grain yield (Prasad *et al*., 2006, 2008*a*; Sánchez *et al.*, 2014; Hatfield and Prueger, 2015). Nevertheless, corn, rice, and sorghum are more tolerant than wheat to high-temperature exposure. For example, although high-temperature conditions after anthesis in both wheat and rice result in smaller grains at maturity, grain size is much more stable at high temperatures in rice than in wheat (Chowdhury and Wardlaw, 1978; Tashiro and Wardlaw, 1989). Furthermore, a comparison of published studies suggests that around anthesis, wheat is sensitive to a lower maximum temperature (32 °C) than both maize and rice (37 °C; Sánchez *et al.*, 2014). Conversely, maize has a shorter phase of anthesis (3–5 d) than wheat, rice, and sorghum (>1 week), which may reduce relative thermotolerance. A longer duration of anthesis reduces the likelihood of a single occurrence of an extreme event affecting all of the flowers (Hatfield and Prueger, 2015). Thus, the duration of anthesis must be considered in the context of projected climate models that predict increases in the incidences of acute high temperatures, and an extended period of anthesis could improve the fertility of wheat under warmer conditions (Lobell *et al.*, 2011).

Taken together, these results suggest that there is potential to transfer knowledge about thermal response mechanisms from maize, rice, and sorghum into wheat and barley to improve their thermotolerance. This approach could be complemented by examining genetic variability for temperature responses among wild barley or wheat (e.g. *Hordeum spontaneum* and *Aegilops* species) that have adapted to warmer growth conditions, which could be utilized in breeding thermotolerant cultivated barley and wheat (Pradhan *et al.*, 2012).

## Molecular mechanisms influencing responses to temperature

**Fig. 2.**
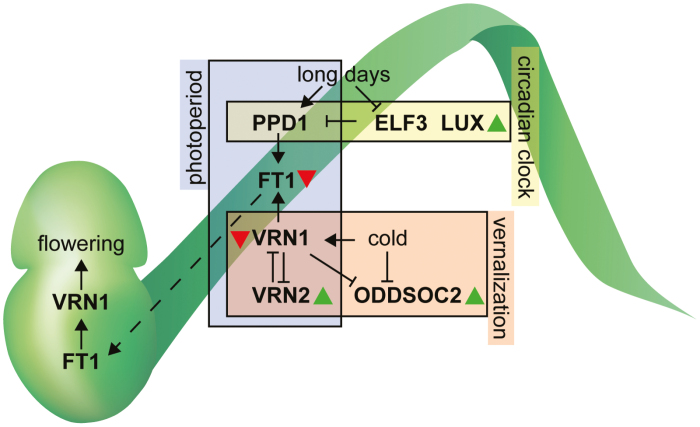
Thermally responsive components of the floral promoting pathway in wheat and barley. Schematic illustrating the interactions between *FT1*, *VRN1*, *VRN2*, *ODDSOC2*, *PPD1*, *ELF3*, and *LUX*, with their reported interactions with high growth temperatures. Boxes indicate genes that are photoperiod dependent, circadian clock regulated, and vernalization responsive. Green arrows indicate up-regulated genes and red arrows indicate down-regulated genes during exposure to high temperatures.

It is essential to understand the molecular mechanisms controlling developmental responses to temperature in order to select or generate varieties with improved tolerance to a warming climate. Many genes involved in the control of developmental responses to photoperiod and vernalization have been identified (Fig. 2; Fjellheim *et al.*, 2014). Flowering in wheat and barley is promoted by long-day photoperiods, with transcriptional activation of *FLOWERING LOCUS T1* (*FT1*) being a central driver of vegetative to floral transition (Yan *et al.*, 2006; Hemming *et al.*, 2008; Lv *et al.*, 2014; Dixon *et al.*, 2018). *ELF3* suppresses flowering under non-inductive photoperiods by indirectly repressing *FT1* expression and gibberellic acid (GA) production, which may occur via the modified activity of *Ppd-1* (Faure *et al.*, 2012; Boden *et al.*, 2014; Gao *et al.*, 2019, Preprint). *Ppd-1* is a pseudo-response regulator that acts downstream of *ELF3* to promote the expression of *FT1*; in wheat, photoperiod-insensitive alleles promote early flowering by misregulating diurnal expression of *Ppd-1*, by either modifying *cis*-regulatory regions or increasing gene copy number, while photoperiod responsiveness is modified in barley by missense alleles of *Ppd-1* that delay flowering (Turner *et al.*, 2005; Beales *et al.*, 2007; Díaz *et al.*, 2012; Shaw *et al.*, 2013; Boden *et al.*, 2015). FT1 protein is expressed in the leaf and translocated to the shoot apical meristem where it interacts with FLOWERING LOCUS D-LIKE (FDL) and 14-3-3 proteins to form the floral activating complex, which induces expression of meristem identity genes, such as the MADS-box transcription factor gene, *VRN1* (Li and Dubcovsky, 2008; Li *et al.*, 2015).

*VRN1* and *VERNALIZATION2* (*VRN2*) are key regulators of the vernalization pathway that activate and repress flowering, respectively. Winter and spring growth types are underpinned by natural variation in *VRN1* and *VRN2* (Danyluk *et al.*, 2003; Trevaskis *et al*., 2003, 2006; Yan *et al*., 2003, 2004). During cold temperatures and short daylengths (winter growth types), *VRN2* expression levels drop, reducing VRN2-mediated repression on *VRN1*, which induces expression of *VRN1* and *FT1* to promote flowering (Yan *et al.*, 2006; Hemming *et al.*, 2008; Li and Dubcovsky, 2008). Spring wheat and barley cultivars contain alleles of *VRN1* that have insertions/deletions in the first intron or mutations in *cis*-regulatory regions, which activate *VRN1* expression in the absence of cold treatment (Danyluk *et al.*, 2003; Trevaskis *et al.*, 2003; Yan *et al.*, 2003). The deletion of *VRN2* also promotes a spring growth habit (Yan *et al.*, 2004; Dubcovsky *et al.*, 2005; Trevaskis *et al.*, 2006). Despite extensive characterization of the mechanisms underpinning low temperature- and photoperiod-dependent reproductive development, insight into the genetic basis of the high temperature responses is more limited.

In the model plant *Arabidopsis thaliana*, the accelerated flowering that occurs under warmer temperatures is underpinned by increased expression of *FT*—the orthologue of wheat and barley *FT1* (Halliday *et al.*, 2003; Balasubramanian *et al.*, 2006). In contrast, analyses in barley have shown that high ambient temperatures repress *FT1* expression, with the absolute transcript levels being dependent on the allelic variation for *ELF3* or *Ppd-1*, or, alternatively, warmer conditions do not influence *FT1* transcript levels (Hemming *et al.*, 2012; Ejaz and von Korff, 2017). Similarly, in spring wheat, warmer ambient temperatures either reduce expression of *FT1* or do not significantly affect *FT1* transcript levels (Kiss *et al.*, 2017; Dixon *et al.*, 2018). Genetic experiments further support *FT1*-independent temperature responsiveness of flowering, with *ft-b1* mutants flowering faster at 24 °C than at 20 °C, as does the wild type (Dixon *et al.*, 2018; Finnegan *et al.*, 2018). Moreover, high *FT1* transcript levels in lines containing daylength-insensitive alleles of *Ppd-1* do not induce rapid early reproductive development at high temperatures in short days (Hemming *et al.*, 2012). In summary, these results suggest that genes or pathways other than the *FT1*-dependent regulation of flowering mediate developmental responses to high temperature in cereals.

In contrast to the photoperiod-dependent pathway, genes involved in vernalization interact strongly with temperature to control flowering time (Kiss *et al.*, 2017; Dixon *et al.*, 2019; Fig. 2). Delayed flowering of winter wheat following exposure to warm temperatures during and after cold treatment genetically maps to *VRN1* (*VRN-A1*; Dixon *et al.*, 2019). Growth at warmer temperatures leads to increased expression of *VRN2* and a MADS-box transcription factor gene that represses flowering, known as *ODDSOC2* (Kiss *et al.*, 2017; Dixon *et al.*, 2019; Fig. 2). The increased expression of *VRN2* is associated with reduced levels of *VRN1* and *FT1* transcripts at warmer temperatures, relative to moderate and cool temperatures (Kiss *et al.*, 2017; Dixon *et al.*, 2019). Genetic analysis showed that allelic variation of *VRN1* disrupts the balance of expression for activators (e.g. *FT1*) and repressors (e.g. *VRN2* and *ODDSOC2*) of flowering in plants grown at 25 °C, relative to 11 °C, with reduced activity of *FT1* delaying flowering and increasing spikelet number when the vernalization requirement has not been satisfied (Greenup *et al.*, 2010; Dixon *et al.*, 2019). Allelic variation for *VRN1* also influences the expression of floral promoting factors in barley, with high-temperature treatments restricting *FT1* expression more strongly in winter lines, relative to spring types, which was associated with stronger repression of *VRN1* at high temperatures in the winter isogenic lines (Ejaz and von Korff, 2017). Similarly, warm growth temperatures inhibit reproductive development under short days but accelerate flowering under long days—the delay under short days coincides with the up-regulation of *ODDSOC2* (Hemming *et al.*, 2012). Taken together, the results highlight a key role for the vernalization pathway in coordinating the developmental response of wheat and barley to growth under warm temperatures.

High temperatures alter the expression of core circadian clock genes, including those for which allelic variation regulates flowering time (Ford *et al.*, 2016; Ejaz and von Korff, 2017; Fig. 2). In barley, the high temperature-dependent increase in transcripts of circadian clock genes *GIGANTEA* (*GI*), *LUX ARRHYTHMO* (*LUX*), and *PSEUDO RESPONSE REGULATOR* (*PRR*) is dependent on *ELF3*, suggesting that *ELF3* may be an essential regulator of photoperiod-dependent flowering during high temperatures (Ford *et al.*, 2016). Under high ambient temperatures, a late-flowering mutant allele of *Ppd-1* delayed floral development and reduced the number of florets and seeds per inflorescence in spring barley. In contrast, floral development occurred earlier, and seed numbers were maintained under high ambient temperatures in both wild-type *Ppd-1* and *elf3* mutants (Ejaz and von Korff, 2017). In wheat, allelic variation for *ELF3* is likely to underpin an *Earliness per se* locus (*Eps-D1*), which interacts with temperature to regulate flowering time, spikelet number, and floret fertility (Lewis *et al.*, 2008; Prieto *et al.*, 2018; Ochagavía *et al.*, 2019). Early-flowering alleles of *Eps-D1* accelerate flowering, reduce spikelet number, and increase floret fertility, particularly when grown at cool temperatures (9–12 °C), while late alleles increase floret fertility at warmer temperatures (18 °C) (Lewis *et al.*, 2008; Prieto *et al.*, 2018; Ochagavía *et al.*, 2019). The variable response of the *Eps-D1* alleles at lower temperatures is potentially dependent on differences in *ELF3* expression, which is significantly different between early and late alleles at 12 °C, but not 18 °C (Ochagavía *et al.*, 2019). These observations suggest that high temperature affects inflorescence development, flowering time, and floret fertility in an *ELF3*- and *Ppd-1*-dependent manner. Given that genetic variation for *Ppd-1* and *ELF3* influences expression of these genes during the evening, it will be interesting to investigate the interaction between early flowering alleles of *Ppd-1* and *ELF3* with high night-time temperatures (Beales *et al.*, 2007; Alvarez *et al.*, 2016).

High temperatures also influence RNA synthesis and protein translation. For example, in the model temperate grass *Brachypodium distachyon*, H2A.Z-nucleosomes play a role in mediating the effects of increased temperature on gene transcription (Boden *et al.*, 2013). H2A.Z-nucleosomes locate to transcription start sites that gate access of the transcriptional machinery into the gene body in a temperature-dependent manner (Raisner *et al.*, 2005; Zhang *et al.*, 2005; Kumar and Wigge, 2010). While H2A.Z levels do not fluctuate in chromatin of leaves as temperature increases, occupancy decreases with rising temperatures in chromatin of developing grain (Boden *et al.*, 2013). The reduced grain weight caused by high temperatures was replicated by genetically perturbing H2A.Z occupancy in chromatin; these results demonstrate a potential mechanism for the reduced grain weight that occurs in wheat and barley under warmer temperatures (Boden *et al.*, 2013).

Regarding translation, protein synthesis *elongation factor Tu* (*EF-Tu*) plays a central role in the elongation phase of protein production (Fu *et al.*, 2008). In wheat, high temperatures increase the accumulation of chloroplast *EF-Tu*, and cultivars expressing greater *EF-Tu* under high-temperature stress display increased thermotolerance (Prasad *et al.*, 2008*b*; Ristic *et al.*, 2008). Following exposure to high-temperature stress, *EF-Tu* exhibits chaperone activity, and heterologous expression of *EF-Tu* decreases the thermal aggregation of leaf proteins and improves heat tolerance (Fu *et al.*, 2008; Prasad *et al.*, 2008*b*). High night-time temperatures increase the expression of *EF-Tu* in wheat, and *EF-Tu* has been implicated in circadian-regulated plant innate immunity in Arabidopsis (Prasad *et al.*, 2008*b*; Bhardwaj *et al.*, 2011; Wang *et al.*, 2011). Taken together, these reports suggest that high temperature-dependent accumulation of *EF-Tu* is important for plant tolerance under thermal stress conditions, potentially in a photoperiod-dependent manner (Fu *et al.*, 2008; Prasad *et al.*, 2008*b*).

## Future directions

### Utilizing genetic diversity and gene editing techniques in cereals

The ability to map genetic traits in wheat and barley has improved dramatically in recent years following the assembly of reference genome sequences for several hexaploid wheat cultivars, tetraploid wheat, diploid progenitor species, and barley (Jia *et al.*, 2013; Krasileva *et al.*, 2017; Luo *et al.*, 2017; Mascher *et al.*, 2017; IWGSC, 2018; Ling *et al.*, 2018). Together with the generation of sequenced mutant and mapping populations, we are now able to identify genes underpinning responses of wheat and barley to higher ambient temperatures (Krasileva *et al.*, 2017; Boden and Østergaard, 2019; Adamski *et al.*, 2020). For example, it is now possible to perform genetic screens using mutant populations to identify genes that confer improved thermal resilience—these screens could take advantage of our deep understanding of traits and developmental stages affected by both chronic and acute heat treatments. Luciferase-based thermal reporters and hyperspectral imaging could help detect genotypes from large populations with modified thermal responsiveness at different developmental stages—this approach has been used productively in Arabidopsis to identify genes that underpin developmental responses to high-temperature treatment (Kumar and Wigge, 2010). These screens could be complemented by an investigation of exotic germplasm grown under semi-arid versus elite conditions, to identify alleles used by landraces or wild progenitors to adapt to warmer conditions. This concept is supported by the D genome progenitor of wheat, *Aegilops tauschii*, which contains more copies of genes involved in abiotic stress and thermal regulation than other major cereals (Jia *et al.*, 2013). Alternatively, small numbers of the crucial domestication alleles could be introduced into the backgrounds of temperature-resilient wild relatives of wheat and barley: a process termed ‘*de novo* domestication’ (Zsögön *et al.*, 2018; Langridge and Waugh, 2019). Such analyses in barley and the diploid progenitors of wheat could help overcome the genetic redundancy that exists in tetraploid and hexaploid wheat, which masks the potential benefit of recessive alleles conferring improved temperature resilience. Alleles identified to improve thermal tolerance could be rapidly introduced into modern cultivars using gene editing such as CRISPR/Cas9 [clustered regularly interspaced palindromic repeats (CRISPR)/CRISPR-associated protein 9] to produce transgene-free genome-edited plants or through use of ‘speed breeding’ (Zhu *et al.*, 2017; Watson *et al.*, 2018).

### Knowledge gained from Arabidopsis could improve our understanding of responses to high temperature in wheat and barley

While our ability to identify genes involved in temperature responses of cereals is improving, there is potential to use the knowledge gained in Arabidopsis to advance our understanding of the molecular pathways that respond to growth under warmer conditions. For example, research has identified multiple genes that contribute to the accelerated flowering of Arabidopsis under chronic warm ambient temperature treatments (e.g. 27 °C versus 17 °C), including *PHYTOCHROME INTERACTING FACTOR4* (*PIF4*), *FLOWERING LOCUS M* (*FLM*), and *SHORT VEGETATIVE PHASE* (*SVP*) (Balasubramanian *et al.*, 2006; Lee *et al.*, 2007; Kumar *et al.*, 2012). *PIF4* expression increases as temperatures rise, and the encoded transcription factor binds to the promoter of *FT* to activate flowering (Kumar *et al.*, 2012). Conversely, SVP interacts with an isoform of FLM (FLM-β) and MADS AFFECTING FLOWERING2 (MAF2) to suppress the expression of floral integrators, including *FT*, *SUPPRESSOR OF CONSTANS* (*SOC1*), *TEMPRANILLO2* (*TEM2*), and *Arabidopsis thaliana CENTRORADIALIS homologue* (*ATC*) (Lee *et al.*, 2007; Posé *et al.*, 2013; Airoldi *et al.*, 2015). High temperatures increase SVP protein degradation, reduce the production of FLM-β, and lower the abundance of the MAF2 isoform that can interact with SVP. Consequently, decreased levels of the SVP:FLM and SVP:MAF2 floral repressor complexes result in the promotion of flowering (Lee *et al.*, 2013; Airoldi *et al.*, 2015). Taken together, these studies highlight the potential that the phytochrome pathway or MADS-box transcription factors that control floral development may be involved in mechanisms regulating high-temperature responses in wheat and barley—indeed, *ODDSOC2* is a MAF-like transcription factor that is more highly expressed under warmer temperatures to repress flowering (Hemming *et al.*, 2012; Dixon *et al.*, 2019). Whether these genes perform conserved roles in cereals remains to be seen; however, the identification of similar mechanisms that either activate or repress flowering would be useful in a breeding context for developing alleles that can fine-tune flowering responses under warmer ambient temperatures. These examples of molecular mechanisms controlling thermal responses in Arabidopsis have focused on the vegetative to floral transition—it would benefit wheat and barley research if future studies using models focused on late developmental stages in order to understand traits that are particularly vulnerable to thermal stress in cereals.

In Arabidopsis and Brachypodium, expression of heat shock protein 70 (HSP70) increases linearly with rising temperatures, identifying the *HSP70* promoter as a potential ‘molecular thermometer’ (Kumar and Wigge, 2010; Boden *et al.*, 2013). Lines containing a fusion of the *HSP70* promoter to luciferase (*HSP::LUC*) have been used in forward genetic screens to identify mutations with altered responses to high temperature (Kumar and Wigge, 2010). Improved transformation capabilities of wheat and barley mean that such strategies could be used to identify genes involved in thermal responses in cereals, including those involved in acute and chronic high-temperature stress. An outcome of this approach in Arabidopsis includes the identification of H2A.Z as an important regulator of the temperature transcriptome in plants (Kumar and Wigge, 2010), suggesting that chromatin-based mechanisms may underpin thermal responses in temperate cereals. The results of this research have been transferred to grasses through work in Brachypodium (Boden *et al.*, 2013); however, the potential for using this mechanism to improve thermal resilience in wheat and barley remains unexplored.

### Modification of developmental and physical traits to enhance thermal resistance

As cereals are most likely to encounter temperature stress during their vulnerable late reproductive stages, modifying the development or thermal resilience of reproductive organs will be vital for maintaining wheat and barley yields under warmer temperatures. For example, rice is relatively tolerant of high temperatures, in part because the panicle architecture helps cool spikelets at the base of the inflorescence relative to those at the apex (Fu *et al.*, 2016). Given that rice forms the majority of grain-producing spikelets at the base of the panicle, the overall yield penalty caused by higher temperatures is relatively low compared with wheat and barley, which produce most of their grain at the centre of the inflorescence. The spike architecture of a wheat and barley inflorescence could be modified to form a panicle-like structure with more spikelets at the base. This arrangement of spikelets may help reduce the adverse effects of warmer temperatures on grain development, which occurs following the emergence of the mature inflorescence. Several genes that regulate inflorescence architecture have been identified in wheat; for example, loss-of-function mutations in the *FRIZZY PANICLE* (*FZP*) gene are associated with an increase of lateral floral meristems, particularly at the base of the inflorescence (Dobrovolskaya *et al.*, 2015; Poursarebani *et al.*, 2015). Further investigation of genes that regulate spikelet arrangements could be used to engineer wheat and barley inflorescences with a higher proportion of florets/spikelets at the base, which may improve the fertility of florets in plants grown at higher temperatures.

Pollen is another reproductive tissue that is sensitive to high-temperature events (Hucl, 1996; Lawrie *et al.*, 2006). Methods that extend the duration of pollination or increase pollen viability could be used to improve the resilience of wheat and barley to warm temperatures. For example, floral development in wheat consists of the primary and secondary openings of the flower, and fully or partially male-sterile lines have a prolonged second opening, which increases the opportunity for cross-pollination (Demotes-Mainard *et al.*, 1996; Okada *et al.*, 2018). Genotypes that produce florets with an extended period of second opening may help buffer the high temperature-dependent loss of viability so that pollen from a neighbouring floret can complete fertilization (Lukac *et al.*, 2012). Alternatively, alleles that improve pollen viability could help enhance fertilization in plants grown at high temperatures. HSPs are involved in heat acclimation and acquired thermal tolerance of developing pollen (Rieu *et al.*, 2017); however, several typical HSPs accumulate less in developing pollen than in vegetative tissue, which may result in increased protein unfolding (Müller and Rieu, 2016). The introduction of HSP alleles with increased expression in pollen may help improve resilience to high-temperature treatments.

The meiotic recombination gene, *DMC1*, has recently been identified as a candidate for high-temperature tolerance during wheat meiosis. Loss of *DMC1* is associated with reduced synapsis and crossovers at 30 °C relative to 20 °C, indicating that DMC1 is required to maintain genome integrity under warmer growth conditions (Draeger *et al.*, 2020). Targeting alleles that counterbalance the adverse effects of high temperatures on grain development could also be a novel strategy for maintaining yield in a warmer climate. Exposure to high temperatures during grain fill results in smaller grains at maturity (Chowdhury and Wardlaw, 1978; Stone and Nicolas, 1995*c*). Introducing alleles, such as the *GW2* locus, that produce larger grains may partially mitigate the negative effect of high temperature on grain size (Simmonds *et al.*, 2016).

## Conclusions

The devastating consequences that warmer temperatures have on cereal yields provides a considerable driver for understanding the mechanisms controlling temperature perception in wheat and barley. The complicated relationship between acute versus constant high-temperature exposure, developmental stage, and time of day at which exposure occurs suggests that the investigation of temperature responses in these cereals will be a fruitful area for fundamental and applied research. Several genes influencing responses to temperature during development have been characterized; however, we have barely scratched the surface of understanding the molecular responses of wheat and barley to warming temperatures. Encouragingly, the emergence of diverse sequenced genomes and gene editing techniques provides hope for rapid improvement in thermal resilience of wheat and barley. This work will be supported by extensive understanding of the physiological response of wheat and barley to warmer temperatures, including the effects of heat on photosynthesis and respiration, and a strong understanding of the molecular mechanisms underpinning similar responses in model organisms.
